# Naphthenic Acid Fraction Components-Induced Metabolic and Mitochondrial Alterations in Rat Hepatoma Cells: Monitoring Metabolic Reprogramming with Tryptophan–Kynurenine Ratio

**DOI:** 10.3390/jox15030061

**Published:** 2025-04-24

**Authors:** Laiba Jamshed, Amica Marie-Lucas, Genevieve A. Perono, Gregg T. Tomy, Jim J. Petrik, Richard A. Frank, L. Mark Hewitt, Philippe J. Thomas, Alison C. Holloway

**Affiliations:** 1 Department of Obstetrics & Gynecology, McMaster University, Hamilton, ON L8S 4L8, Canada; jamshel@mcmaster.ca (L.J.);; 2 Centre of Oil and Gas Research and Development, University of Manitoba, Winnipeg, MB R3T 2N2, Canadagregg.tomy@umanitoba.ca (G.T.T.); 3 Department of Biomedical Sciences, University of Guelph, Guelph, ON N1G 2W1, Canada; 4 Water Science and Technology Directorate, Environment and Climate Change Canada, Burlington, ON L7S 1A1, Canada; richard.frank@ec.gc.ca (R.A.F.);; 5 Wildlife and Landscape Science Directorate, Environment and Climate Change Canada, National Wildlife Research Center, Ottawa, ON K1A 0H3, Canada

**Keywords:** AOSR, NAFC, mitochondrial dysfunction, metabolic reprogramming, metabolic switch, substrate utilization, KTR, tryptophan metabolism, kynurenine

## Abstract

Altered body condition and diminished growth in wildlife in the Alberta Oil Sands Region (AOSR) are prompting investigations into the impact of oil sands industrial activity on wildlife in the region. Chemical constituents from bitumen-influenced waters, including oil sands process-affected water (OSPW), can disrupt endocrine signaling, leading to aberrant lipid accumulation and altered glycemic control in mammals. This study aimed to investigate the effects of naphthenic acid fraction components (NAFCs), derived from OSPW, on energy homeostasis using the McA-RH7777 rat hepatocyte model. Cells were exposed to NAFCs at nominal concentrations of 0, 0.73, 14.7, and 73.4 mg/L for 24 and 48 h. We assessed gene expression related to lipid and glucose metabolism and measured triglyceride accumulation, glucose, and fatty acid uptake. NAFC exposure (14.7 and 73.4 mg/L) reduced triglyceride levels and glucose uptake and increased fatty acid uptake and the expression of beta-oxidation genes, suggesting a metabolic switch from glucose to fatty acid oxidation. This switch in substrate availability signifies a shift in cellular energy dynamics, potentially linked to altered mitochondrial function. To investigate this, we conducted adenosine triphosphate (ATP), mitochondrial membrane potential, and terminal deoxynucleotidyl transferase dUTP nick-end labeling (TUNEL) assays to measure cellular ATP levels, mitochondrial membrane potential, and apoptosis, respectively. At both time points, 73.4 mg/L NAFC exposure resulted in increased ATP levels, induced mitochondrial membrane hyperpolarization, and increased apoptosis. These results suggest that mitochondrial efficiency is compromised, necessitating metabolic adaptations to maintain energy homeostasis. Given that cells exhibit metabolic flexibility that allows them to dynamically respond to changes in substrate availability, we further demonstrated that the kynurenine–tryptophan ratio (KTR) serves as a marker for a shift in energy metabolism under these stress conditions. This work provides a mechanistic framework for understanding how bitumen-derived organic contaminants may disrupt metabolic function in wildlife living in the AOSR. These findings further support the use of molecular markers like KTR to evaluate sub-lethal metabolic stress in environmental health monitoring.

## 1. Introduction

As the third largest oil reserve in the world, the Alberta Oil Sands Region (AOSR) is a major source of revenue, energy, and economic success for both the province and Canada. Comprising three regions—Athabasca, Cold Lake, and Peace River—these oil sands deposits consist of a mixture of sand, clay, water, and bitumen. The extraction and processing of bitumen from these reserves has provided numerous jobs, spurred infrastructure development, and bolstered Canada’s position in the global energy market. However, alongside these economic benefits, continued industrial development in the AOSR has sparked significant environmental and health concerns.

A by-product of the extraction process of bitumen results in the production and storage of oil sands process-affected water (OSPW) in tailings ponds [1,2,3,4]. The tailings ponds in northern Alberta, store ~1.4 trillion liters of OSPW. A “zero discharge” policy is in place to prevent the release of tailings water into the environment [5]; however, reports of numerous leaks and spills [6] leading to localized contamination have resulted in increased concerns about the effectiveness of tailings management. In recent years, discussions and proposals have emerged considering the eventual release of treated tailings pond water into the Athabasca River system in northern Alberta. Located downstream of oil sands industrial operations, the Peace–Athabasca Delta (PAD) is recognized as the world’s largest inland boreal delta [7]. The PAD supports large numbers of migratory birds, species at risk (e.g., wood bison and whooping cranes), and countless other species. The PAD is recognized as a Key Biodiversity Area, an RAMSAR wetland, and part of the Outstanding Universal Value of the UNESCO-recognized Wood Buffalo National Park World Heritage Site. Moreover, this region supports the livelihoods and cultural practices of Indigenous communities in the region [8]. As OSPW and its constituents have been shown to be toxic to various species, the efficacy of treatment strategies employed prior to the potential reintroduction of treated-OSPW into the environment is a significant concern for scientists, policy makers and peoples living in the region.

While the composition of OSPW varies depending on several factors including (i) host ore spatial differences, (ii) the method of the extraction (i.e., open-pit mining, in situ recovery, solvent extraction), (iii) the geological location of the mine, and (iv) the duration of OSPW storage in the tailings ponds, it is evident that certain contaminants are consistently present across all OSPW samples [3]. These include elevated levels of salts, specific metals, polycyclic aromatic compounds (PACs), BTEX (benzene, toluene, ethylbenzene, and xylenes), and dissolved organic compounds, including naphthenic acid fraction components (NAFCs) [6,7,8,9,10,11]. NAFCs are naturally occurring in bitumen and are therefore present in the Athabasca River watershed flowing through the oil sands deposits. However, they become highly concentrated in OSPW due to the extraction processes [12,13] and can enter the environment through leachates from tailings ponds and be transported via groundwater flow paths in combination with naturally leached bitumen [1,3,14,15,16,17].

Many studies have identified NAFCs as a key driver of OSPW toxicity. Chronic exposure to NAFCs has been found to cause reproductive, developmental, and behavioral abnormalities in various aquatic species, including fish, amphibians, and invertebrates [4,8,18]. While much of the research has focused on aquatic organisms, the potential implications for mammalian health are a growing concern, particularly for wildlife populations in the PAD [19]. Recently, studies have shown that NAFCs can disrupt endocrine signaling and impair metabolic health, leading to abnormal lipid accumulation and changes in glucose regulation [2,20]. Importantly, changes in glucose and lipid availability are often linked to altered energy homeostasis including mitochondrial dysfunction and amino acid metabolism [21,22]. While NAFCs have been shown to disrupt glucose and lipid homeostasis in non-mammalian vertebrates, their effects on mammalian metabolic homeostasis, including mitochondrial function and amino acid metabolism, are unknown and are the focus of this study.

Cellular resource allocation is central to how cells manage nutrient availability, bioenergetic capacity, and macromolecule production, all of which are crucial to maintaining metabolic homeostasis [23]. Hepatocytes, the liver cells responsible for regulating glucose, lipid, and protein metabolism, are functional indicators of this balance and are key to understanding how organisms adapt to their microenvironments. In this study, we investigated the effects of NAFC exposure on metabolic homeostasis using McA-RH7777 cells, a rat hepatocyte model of dyslipidemia. The cells were exposed to NAFCs at concentrations reflective of environmental exposure levels [24,25,26,27,28,29] [0, 0.73, 14.7, 73.4 mg/L] for 24 and 48 h to determine how these bitumen-derived dissolved organics influence energy homeostasis in a mammalian system.

## 2. Materials and Methods

### 2.1. Preparation and Storage of NAFCs

Preparation of the NAFC stock solution has been previously described (Frank et al., 2006) [11]. Briefly, ~2000 L of OSPW was collected in 2009 from an active tailings pond at Industry A and stored at 4 °C in the dark. Extraction and purification of NAFCs commenced within six months of sample collection [11]; this was followed by quantification according to the method described by Brunswick et al. (2015) [30] and chemical characterization as outlined in Marentette (2015) [24]. The NAFC extracts were stored in amber bottles at 4 °C for all exposures.

### 2.2. Cell Culture Maintenance and Treatment

McA-RH7777 cells are an immortalized rat hepatoma cell line widely used as a well-established model of dyslipidemia and hepatic metabolic dysfunction. The McA-RH7777 cell line was obtained from ATCC (CRL-1601; Manassas, VA, USA). Due to their intact lipid and glucose metabolic pathways and sensitivity to endocrine disruption, they serve as a relevant in vitro system for investigating how environmental toxicants affect hepatic energy metabolism and mitochondrial function [31,32,33].

McA-RH7777 cells were cultured at 37 °C in a humidified atmosphere of 95% O_2_ and 5% CO_2_ in Dulbecco’s Modified Eagle Medium (DMEM 1X with 4.5 g/L glucose, L-glutamine, and sodium pyruvate; Corning, Manassas, VA, USA), supplemented with 10% (*v*/*v*) fetal bovine serum (FBS; Gibco, Grand Island, NY, USA), 2 mM L-glutamine, 100 U/mL penicillin, and 100 μg/mL streptomycin (Gibco). Unless otherwise noted, experimental protocols were carried out in DMEM media supplemented as described.

McA-RH7777 cells were cultured in 10 mm dishes (Corning), with fresh medium replaced every 48 h. Sub-culturing was performed once cells reached ~80% confluence and exhibited characteristic spindle-like morphology. Confluency was defined as 80% plate coverage following a minimum of two passages. All experiments were conducted using confluent cells from passages 11 to 13. For assays, cells were seeded in a 96-well, flat-bottom tissue-culture-treated microplates (Corning) at a density of 150,000 cells/well for 24 h exposure and 100,000 cells/mL for 48 h exposure. After 24 h, cells were treated with NAFC-supplemented media for 24 or 48 h. All functional assays were carried out using cells between passages 12 and 13, as described in the sections below.

To investigate gene expression via dose–response exposure experiments, confluent McA-RH7777s from passage 11 were seeded at a density of 200,000 cells/mL in 6-well plates (Corning) for 24 h exposure and 150,000 cells/mL for 48 h exposure. The cells were allowed to reach ~85% confluency prior to treatment. The cells were the exposed to vehicle control (DMEM) or NAFCs [0.73, 14.7, and 73.4 mg/L] for 24 and 48 h in supplemented DMEM, as previously described (N = 6 independent experiments) [11]. The selected NAFC concentrations [0.73, 14.7, and 73.4 mg/L] reflect environmentally relevant levels reported in surface waters, wetlands, tailings ponds, and OSPW from the Athabasca Oil Sands Region [24,25,26,27,28,29].

### 2.3. qPCR

Quantitative polymerase chain reaction (qPCR) was used to assess changes in the mRNA expression of genes involved in cellular metabolic responses to environmental stresses. These included genes associated with lipid and glucose metabolism, cellular stress response, and tryptophan metabolism. The genes involved in lipid metabolism include *Fasn* (fatty-acid synthase) and *Acca* (Acetyl-CoA carboxylase alpha), which are crucial for fatty acid synthesis; *Cpt1α* (carnitine palmitoyltransferase 1 alpha), which facilitates the transport of fatty acids into the mitochondria for beta-oxidation; and *Cd36* (fatty acid translocase), which is involved in fatty acid uptake. For glucose metabolism, *G6pase* (glucose-6-phosphatase), the enzyme essential for the final steps of gluconeogenesis and glycogenolysis, was assessed. *Pgc1a* (peroxisome proliferator-activated receptor-γ coactivator), a key regulator of genes involved in energy metabolism and mitochondrial biogenesis was also assessed.

Total RNA was isolated from treated cells using TRIzol reagent (Invitrogen, Carlsbad, CA, USA). RNA concentration and quality were determined using the NanoDrop One^TM^ Microvolume UV–Vis Spectrophotometer (Thermo Scientific, Waltham, MA, USA). Complementary DNA (cDNA) was synthesized from extracted RNA using the High-Capacity cDNA Reverse Transcription Kit (Applied Biosystems, Foster City, CA, USA) as per the manufacturer’s instructions. RT-qPCR was performed using PerfeCta^®^ SYBR^®^ green FastMix^®^ (Quantabio, Beverly, MA, USA) on the CFX384 Touch^TM^ Real-Time PCR Detection System (Bio-Rad, Hercules, CA, USA). The cycling conditions included polymerase activation (95 °C for 10 min), followed by 40 cycles of denaturing (95 °C for 10 s), annealing (60 °C for 10 s), and extension (72 °C for 15 s, slow-ramp rate of 2.5 °C per s). Relative gene expression was determined using the comparative ΔΔCt method [34], with data normalized to the geometric mean expression of the reference genes: beta-2-microglobulin (*B2m*) and peptidylprolyl isomerase A (*Ppia*). The primer sequences used are presented in Table 1.

### 2.4. Metabolic Function

To assess whether NAFC-induced transcriptomic alterations in key hepatic metabolic pathways resulted in measurable functional changes, we quantified glucose uptake, triglyceride levels, and fatty acid uptake.

*Glucose Uptake Assay:* Glucose uptake was measured using a Glucose Uptake-Glo^TM^ Assay (Promega) according to the manufacturer’s protocol (N = 10). Briefly, McA-RH7777 cells were plated in 96-well plates and treated with the vehicle control or NAFCs [0.73, 14.7, and 73.4 mg/L] for 24 and 48 h (N = 10 independent experiments). Luminescence was measured using a Synergy H1 Hybrid Reader (Agilent Technologies, Inc.).

*Triglyceride Assay:* To determine the effects of NAFCs on triglyceride content, a Triglyceride-Glo^TM^ Assay (Promega, Madison, WI, USA) was performed according to the manufacturer’s protocol (N = 9 independent experiments). Briefly, McA-RH7777 cells were plated in 96-well plates and treated with the vehicle control or NAFCs [0.73, 14.7, and 73.4 mg/L] for 24 and 48 h. Luminescence was measured using a Synergy H1 Hybrid Reader (Agilent Technologies, Inc., Santa Clara, CA, USA). Triglyceride concentrations were quantified against an 8-point standard curve.

*Fatty Acid Uptake Assay:* To determine the effects of NAFCs on fatty acid uptake, a Fatty Acid Uptake Assay Kit (Abcam, Cambridge, UK) was used according to the manufacturer’s protocol (N = 5 independent experiments). Briefly, McA-RH7777 cells were plated in 96-well plates and treated with the vehicle control or NAFCs [0.73, 14.7, and 73.4 mg/L] for 24 h and 48 h, in serum-free, phenol red-free DMEM. Fluorescence measurements (Ex/Em = 488/523 nm) were conducted in kinetic mode at 37 °C for 60 min, followed by endpoint florescence in bottom-read mode, using a Synergy H1 Hybrid Reader (Agilent Technologies, Inc.).

### 2.5. Mitochondrial Function

Proper mitochondrial function is crucial for energy production and cellular integrity and viability. Disruptions in mitochondrial activity can lead to significant metabolic and bioenergetic challenges, impacting cell survival and function. In this study, we employed two assays to assess mitochondrial health in McA-RH7777 cells following exposure to NAFCs for 24 and 48 h.

*ATP Assay:* To assess the effects of NAFCs on ATP production, a key indicator of mitochondrial performance and intracellular energy homeostasis, we used a Luminescent ATP Detection Assay Kit (Abcam) according to the manufacturer’s protocol (N = 6 independent experiments). Briefly, McA-RH7777 cells were plated in 96-well plates and treated with the vehicle control or NAFCs [0.73, 14.7, and 73.4 mg/L] for 24 and 48 h. Luminescence was measured using a Synergy H1 Hybrid Reader (Agilent Technologies, Inc.).

*JC-1 Assay:* To assess the effects of NAFCs on mitochondrial membrane potential (Δψm), a JC-1 Assay (Abcam) was performed according to the manufacturer’s protocol (N = 8 independent experiments). Briefly, cells were seeded in 96-well plates and allowed to adhere for 24 h, after which the cells were exposed to the vehicle control or NAFCs [0.73, 14.7, and 73.4 mg/L] for 24 and 48 h. Fluorescence measurements were taken for JC-1 aggregates and monomers (Ex/Em = 475/590, 530 nm) on a Synergy H1 Hybrid Reader (Agilent Technologies, Inc.).

### 2.6. Apoptosis

To determine if NAFC exposure caused increased cell death via apoptosis, terminal deoxynucleotidyl transferase dUTP nick-end labeling (TUNEL) staining was performed with a DeadEnd fluorometric TUNEL system (Promega) following the manufacturer’s protocol [35]. Briefly, the cells were seeded in chamber slides (N = 4 independent experiments) and treated with the vehicle control or NAFCs [0.73, 14.7, and 73.4 mg/L] for 24 and 48 h. Positive (DNase I) and negative controls were used according to the kit recommendations. Stained slides were imaged, and TUNEL-positive cells were quantified using Image J software (v1.54j, National Institutes of Health, Bethesda, MD, USA). The percentage of TUNEL-positive cells per section was calculated and averaged across treatment groups.

### 2.7. Amino Acid Metabolism

NAFCs have been found to cause oxidative stress, mitochondrial dysfunction, and cell cycle dysregulation, processes that have all been linked to metabolic perturbations and changes in amino acid pathways [36]. Tryptophan is an essential amino acid involved in numerous physiological processes, including protein accretion (growth), neurotransmitter synthesis, regulation of mood and sleep, immune function, and gastrointestinal health. Tryptophan is primarily catabolized through the kynurenine pathway (Trp-Kyn), but it is also the precursor for serotonin and melatonin. There are now a number of studies that have shown that tryptophan catabolism is altered in association with perturbations in glucose and lipid homeostasis [37,38,39]. Moreover, non-lethal endpoint toxicity studies across species have shown that NAFC exposure can affect aryl hydrocarbon receptor (AhR) signaling, glucocorticoid receptor (GR) signaling, oxidative stress, and inflammatory pathways [40,41,42,43,44,45,46,47], all of which have been reported to regulate the Trp-Kyn pathway. Therefore, we assessed changes in Trp, Kyn, and the kynurenine–tryptophan (KTR) ratio following NAFC exposure [48].

*Materials and reagents:* Unlabeled analytical standards of L-tryptophan (TRP, ≥98% HPLC grade) and L-kynurenine (KYN, ≥98%) were obtained from Sigma-Aldrich Canada Co. (Oakville, ON, Canada). Stable isotope-labeled standards, L-tryptophan-2′,4′,5′,6′,7′-d5 (indole-d_5_) (98%, TRP-d_5_), and L-kynurenine-d_4_ [4-(2-aminophenyl-3,5-d_2_)] (99%, KYN-d_4_) were purchased from CDN Isotopes (Pointe-Claire, QB, CA). All solutions were stored at 4 °C. L-Acetonitrile (ACN) and water were purchased from Fisher-Scientific and were of HPLC grade. Ultrapure Milli-Q was obtained using a Synergy^TM^ Milli-Q purification system from Millipore (Billerica, MA, USA).

*Preparation of samples for chromatographic analysis:* To a 2 mL glass vial, 60 μL of in vitro media and TRP-d_5_ and KYN-d_4_ (20 μL of a mixture containing 7.5 ng/μL each) were added and adjusted to a final volume of 1.5 mL with ACN (1420 μL). Samples were vortexed for 30 secs prior to chromatographic analyses. Method blanks consisting only of the control media were prepared in an identical manner to our samples. Reagent blanks of ACN were injected periodically to assess the extent of contamination from the solvent and between injections. Matrix effects were assessed by fortifying control media (60 μL) with our native and mass-labeled compounds (20 μL each and diluting to a final volume of 1.5 mL) and comparing their signals to that of an external standard prepared in ACN. Limits of detection and quantitation were determined according to Eurachem guidelines by fortifying control media with native TRP and KYN (n = 6).

*High-Performance Liquid Chromatography–Tandem Mass Spectrometry:* The detection and quantitation of TRP and KYN in biological matrices was based on a previously published validated method [49]. Separations of mass-labeled and native KYN and TRP were performed using a SEQUANT ZICR HILIC PEEK-coated HPLC column (4.6 mm × 100 mm, 3.5 μM particle size, 200 A pore size) (Merck, Darnstadt, Germany). The HPLC was coupled to a Sciex 365 triple quadrupole mass spectrometer retrofitted with an HSID Ionics EP+ orthogonal ionization source. Ionization was performed in the positive ion mode, and the multiple reaction monitoring ion transitions used for detection and quantitation were as follows: TRP: *m*/*z* 205.1 → 188.2, KYN: *m*/*z* 209.4 → 94.1, TRP-d_5_: *m*/*z* 210.2 → 192.4, and KYN-d_4_: *m*/*z* 213.3 → 196.2. No statistical differences (*p* < 0.05) were observed between the response of the target analytes in media relative to the standards prepared in solvent, implying that matrix effects were negligible. The limits of detection and quantitation for TRP were 2.8 and 9.5 pg/μL and those for KYN were 4.3 and 14.3 pg/μL, respectively.

Total naphthenic acids were quantified following the method of Brunswick et al. (2015) [30]. Samples were adjusted to pH 10 with direct injection into an Agilent Infinity 1290 HPLC system fitted to an Agilent 6550 iFunnel AJS Series Liquid Chromatograph Mass Spectrometer with a Time-of-Flight detector (LC/MS-ToF). Analytes were detected by total ion scan (TIC) from *m*/*z* 110 to 990 and utilized a library of over 400 C_9_–C_30_ isomers. Instrument calibrations utilized a Merichem technical naphthenic acid mixture.

### 2.8. Statistical Analysis

All statistical analyses were conducted using GraphPad Prism (v.9.5.1, GraphPad Software, San Diego, CA, USA). Data were first assessed for outliers using Grubb’s Test, tested for normality with the Shapiro–Wilk test, and checked for equal variance. For outcome measures requiring comparisons between control and multiple treatment groups, a one-way ANOVA was performed, followed by Dunnett’s post hoc tests when significant differences were detected (*p* ≤ 0.05). For data that did not pass tests for normality or equal variance, a Kruskal–Wallis one-way ANOVA on ranks was used, with Dunn’s post hoc test to compare the treatment groups with the control. Gene expression data were analyzed using the ΔΔCt method [34], which includes normalization of the cycle thresholds of the target gene to housekeeping genes (*B2m* and *Ppia*), followed by normalization to the control group and a final transformation using 2^(−ΔΔCt)^ to calculate relative fold changes. These values were then analyzed using one-way ANOVA (or Kruskal–Wallis if nonparametric), followed by the appropriate post hoc test as described above. All results are presented as mean ± SEM and were considered significant when *p* ≤ 0.05.

## 3. Results

### 3.1. Exposure to 14.7 and 73.4 mg/L NAFCs Induces Significant Metabolic Shifts in McA-RH7777 Cells

#### 3.1.1. Glucose

**Figure 1:**
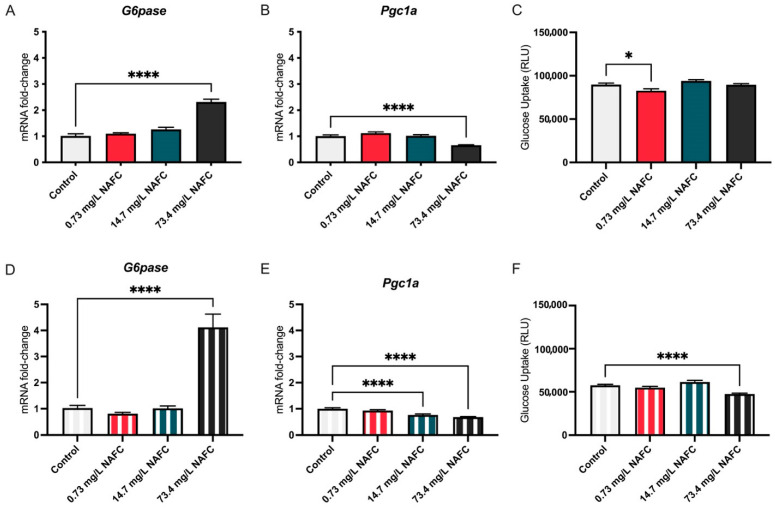
mRNA expression of gluconeogenesis pathway genes G6pase (**A**,**D**) and Pgc1a (**B**,**E**) and glucose uptake (**C**,**F**) in McA-RH7777 cells following 24 h (colored bars) and 48 h (striped bars) exposure to NAFCs [0, 0.73, 14.7, 73.4 mg/L]. Data are presented as mean ± SEM (N = 6 genes; N = 10 glucose uptake assay). Statistical significance compared to control is denoted by asterisks: * *p* < 0.05, **** *p* < 0.001.

NAFC exposure led to time- and dose-dependent alterations in the expression of gluconeogenesis-related genes and glucose uptake in McA-RH7777 cells. At 24 h, treatment with 73.4 mg/L NAFCs resulted in a significant increase in *G6pase* mRNA expression (Figure 1A), while *Pgc1a* expression was notably decreased (Figure 1B). By 48 h, the increase in G6pase expression at 73.4 mg/L was even more pronounced (Figure 1D), and *Pgc1a* expression was significantly reduced at both 14.7 mg/L and 73.4 mg/L (Figure 1E). In parallel, the glucose uptake assay revealed that at 24 h, there was no significant change in glucose uptake at 73.4 mg/L NAFCs, but a small and significant reduction was observed at the lower dose of 0.73 mg/L (Figure 1C). However, by 48 h, glucose uptake was significantly decreased at the 73.4 mg/L concentration (Figure 1F).

#### 3.1.2. Lipids

**Figure 2:**
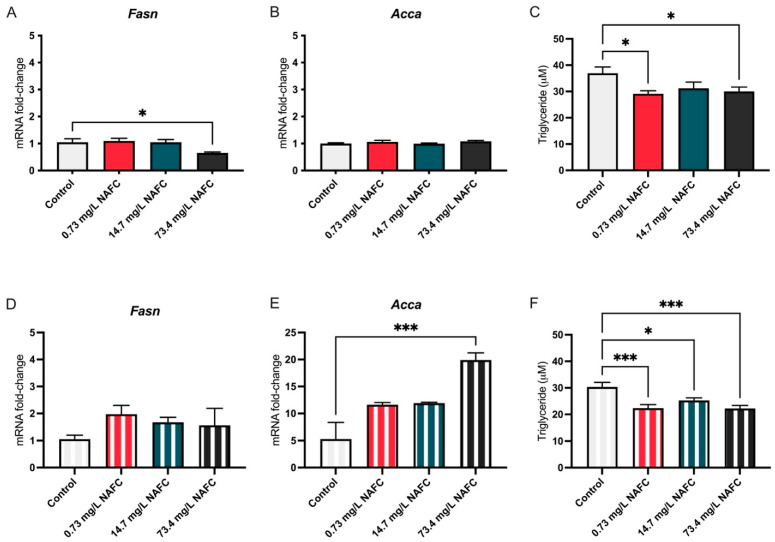
mRNA expression of de novo lipogenesis pathway genes Fasn (**A**,**D**) and Acca (**B**,**E**) and triglyceride accumulation (**C**,**F**) in McA-RH7777 cells after 24 h (colored bars) and 48 h (striped bars) exposure to NAFCs [0, 0.73, 14.7, 73.4 mg/L]. Data are presented as mean ± SEM (N = 6 genes; N = 9 triglyceride assay). Statistical significance compared to control is denoted by asterisks: * *p* < 0.05, *** *p* < 0.005.

NAFC exposure induced time- and dose-dependent changes in both gene expression and triglyceride content in McA-RH7777 cells. At 24 h, a significant decrease in Fasn mRNA expression was observed with 73.4 mg/L NAFCs (Figure 2A), which corresponded with a reduction in triglyceride levels at the same concentration (Figure 2C). Interestingly, at 48 h, while 73.4 mg/L NAFCs reduced triglyceride levels (Figure 2F), it also significantly increased Acca mRNA expression (Figure 2E). Notably, at 48 h, triglyceride levels were decreased across all tested doses of NAFCs (Figure 2F).

**Figure 3:**
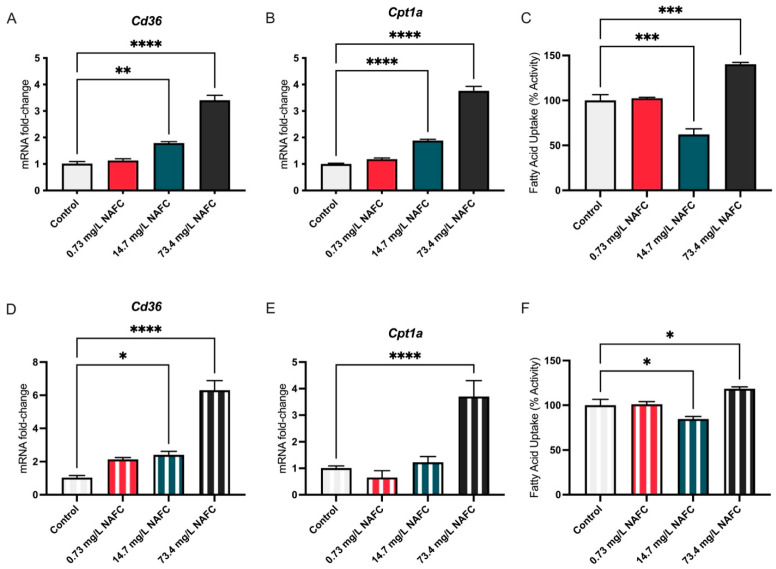
mRNA expression of fatty acid oxidation pathway genes Cd36 (**A**,**D**) and Cpt1α (**B**,**E**) and fatty acid uptake (**C**,**F**) in McA-RH7777 cells following 24 h (colored bars) and 48 h (striped bars) exposure to NAFCs [0, 0.73, 14.7, 73.4 mg/L]. Data are presented as mean ± SEM (N = 6 genes; N = 5 fatty acid uptake assay). Statistical significance compared to control is denoted by asterisks: * *p* < 0.05, ** *p* < 0.01, *** *p* < 0.005, **** *p* < 0.001.

The expression of *Cd36*, a key regulator of fatty acid uptake, was significantly increased at both 14.7 mg/L and 73.4 mg/L NAFCs at 24 and 48 h (Figure 3A,D). Similarly, *Cpt1α* expression increased at both 14.7 mg/L and 73.4 mg/L at 24 h (Figure 3B), but by 48 h, this increase was observed only at the higher dose of 73.4 mg/L (Figure 3E). These gene expression changes translated into distinct functional outcomes in fatty acid uptake. At both 24 and 48 h, the 14.7 mg/L dose resulted in decreased fatty acid uptake (Figure 3C), despite the upregulation of *Cd36*. In contrast, at the higher dose of 73.4 mg/L, fatty acid uptake was significantly increased at both time points (Figure 3F).

### 3.2. Metabolic Shifts in NAFC-Exposed McA-RH7777 Cells Suggest Alterations in Mitochondrial Function

**Figure 4:**
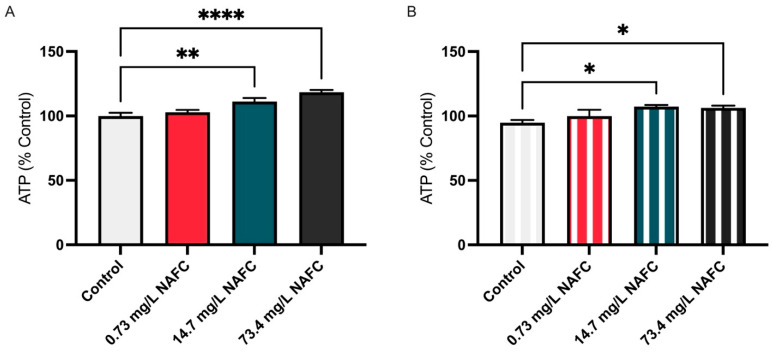
ATP levels in McA-RH7777 cells following exposure to NAFCs [0, 0.73, 14.7, 73.4 mg/L] measured at (**A**) 24 and (**B**) 48 h. Data are presented as mean ± SEM (N = 6). Statistical significance compared to control is denoted by asterisks: * *p* < 0.05, ** *p* < 0.01, **** *p* < 0.001.

NAFC exposure also had a significant impact on cellular energy production, as indicated by changes in ATP levels. Across both 24 and 48 h of exposure, ATP levels were significantly increased in McA-RH7777 cells (Figure 4).

**Figure 5:**
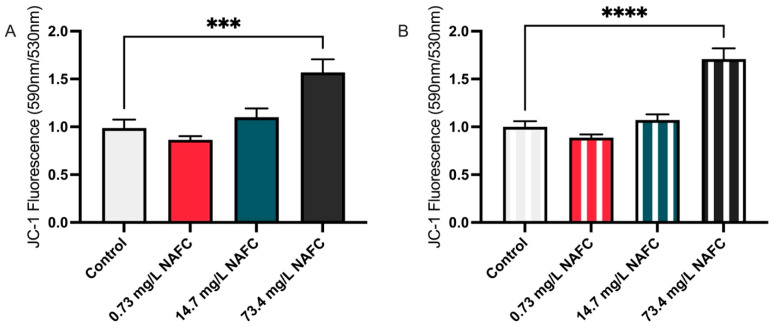
Mitochondrial membrane potential in McA-RH7777 cells assessed by JC-1 assay following (**A**) 24 h and (**B**) 48 h exposure to NAFCs [0, 0.73, 14.7, 73.4 mg/L]. Fluorescence intensity ratios of JC-1 aggregates to monomers were quantified, indicating changes in mitochondrial health. Data are presented as mean ± SEM (N = 8). Statistical significance compared to control is denoted by asterisks: *** *p* < 0.005, **** *p* < 0.001.

In addition to changes in ATP levels, we assessed mitochondrial membrane potential (Δψm) using the JC-1 assay. Exposure to 73.4 mg/L NAFCs resulted in a significant increase in JC-1 fluorescence at both 24 and 48 h (Figure 5).

**Figure 6:**
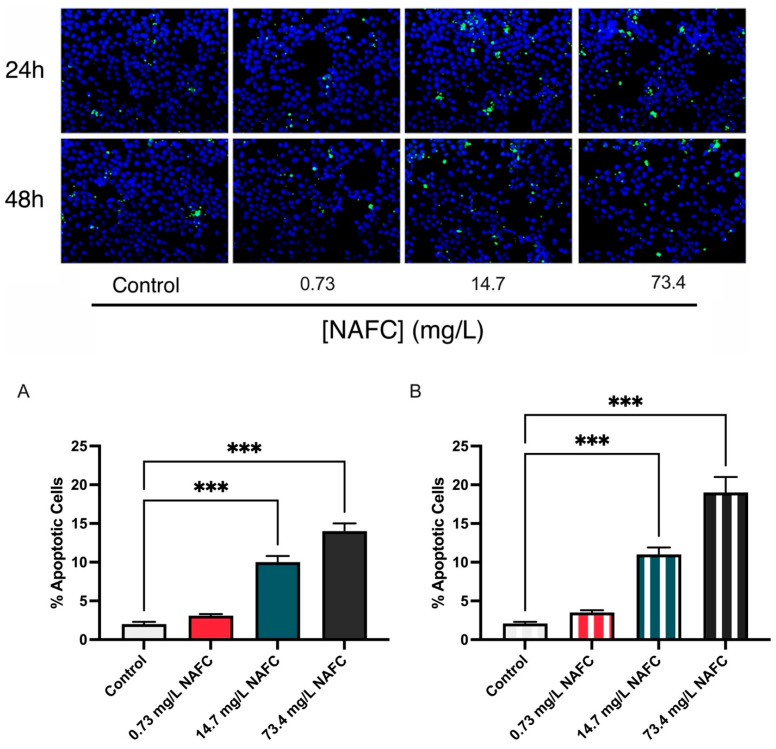
Apoptotic cell detection in McA-RH7777 cells using the TUNEL assay after 24 h (**A**) and 48 h (**B**) exposure to NAFCs [0, 0.73, 14.7, 73.4 mg/L]. TUNEL-positive cells were quantified to assess the extent of apoptosis induced by the treatment. Data are presented as mean ± SEM for four replicates (N = 4). Statistical significance compared to control is denoted by asterisks: *** *p* < 0.005.

This increase in mitochondrial ATP production and Δψm was accompanied by increased apoptosis, as measured by the TUNEL assay. Both 14.7 mg/L and 73.4 mg/L NAFCs induced a significant increase in TUNEL-positive cells at both time points (Figure 6).

### 3.3. The Kynurenine–Tryptophan Ratio Was Altered in Association with Changes in Substrate Utilization and Metabolic Alterations in McA-RH7777 Cells Following Exposure to 73.4 mg/L NAFCs

**Figure 7:**
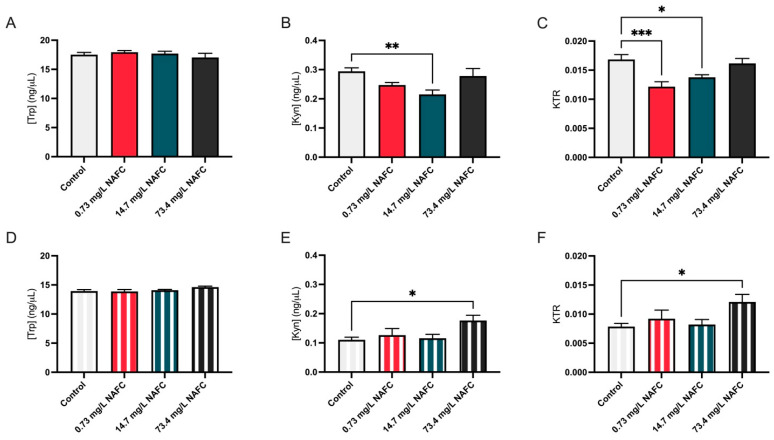
TRP levels (**A**,**D**), KYN levels (**B**,**E**), and KTR (**C**,**F**) in McA-RH7777 cells following 24 h (colored bars) and 48 h (striped bars) exposure to NAFCs [0, 0.73, 14.7, 73.4 mg/L]. Data are presented as mean ± SEM (N = 6). Statistical significance compared to control is denoted by asterisks: * *p* < 0.05, ** *p* < 0.01, *** *p* < 0.005.

Lastly, we assessed the impact of NAFC exposure on the Trp-Kyn pathway. NAFC exposure led to higher KYN levels and an elevated KTR at the 48 h time point following exposure to 73.4 mg/L NAFCs (Figure 7E,F). Interestingly, at 24 h, the 14.7 mg/L dose led to a decrease in KYN levels, and both 0.73 mg/L and 14.7 mg/L doses resulted in a decreased KTR, despite no significant changes in TRP or KYN levels at the 0.73 mg/L dose (Figure 7B,C).

## 4. Discussion

The toxicity of OSPW and its constituents, particularly NAFCs, remains largely unexplored in mammals. Given the critical role that metabolic processes play in organismal health and survival, it is essential to understand how exposure to these chemical components might be disrupting energy homeostasis and, in turn, mammalian health. The present study used an NAFC extract consistent with previous laboratory studies on exposure effects [24,25,41,43,49]. In the 2024 reanalysis of this NAFC extract for the present study, the Merichem technical mixture served as the instrument calibrant, selected for its role as a traceable reference material. In earlier studies, concentration ranges were based on previous calibration standards, with Merichem now providing a reliable, updated calibration reference. Using the same NAFC extracts, we previously reported significant changes in mammalian bone metabolism at concentrations ranging from 12.5 to 125 mg/L [43]. With the most recent analysis of the concentration in this mixture, these results correspond to 7.34–73.4 mg/L, consistent with levels found in wetlands impacted by tailings seepage and groundwater contamination [17,29].

### 4.1. Metabolic Substrate Utilization in Response to NAFC Exposure

NAFC exposure led to significant metabolic alterations in McA-RH7777 cells. Specifically, exposure to 73.4 mg/L NAFCs resulted in a marked decrease in triglyceride levels and glucose uptake at both 24 and 48 h. These changes were accompanied by decreased expression of the lipogenesis genes, *Fasn* and *Pgc1α*, suggesting a suppression of glucose-driven lipid synthesis. These findings are consistent with previous research on invertebrates, where NAFCs were shown to disrupt metabolic processes, leading to altered lipid profiles (reviewed in [2,20]). In aquatic species, naphthenic acids (NAs) have been shown to reduce metabolic activity in rainbow trout (*Oncorhynchus mykiss*) hepatocytes [50] and disrupt liver and cholesterol metabolism in frog embryos. Exposure to NA in *Lithobates pipiens* tadpoles resulted in impaired growth and development, diminished glycogen reserves, and elevated triglyceride levels, indicating significant disruptions in energy metabolism and hepatic glycolysis [50]. Similarly, frog *Silurana* (*Xenopus*) *tropicalis* embryos exposed to NA had reduced body size and perturbations in liver and cholesterol metabolism [40].

While the adverse effects of NAFCs on aquatic life are well documented, less is known about their impact on mammalian and terrestrial wildlife [51,52]. Recent observations of altered body condition and diminished growth in wildlife within the AOSR suggest that these contaminants may similarly affect terrestrial species [51,52,53,54,55]. In the limited studies that do exist, there is evidence that NAFCs can adversely affect metabolic homeostasis. Rogers et al. (2002) [56] exposed rats to an organic fraction of acidified OSPW, which included NAFCs and low amounts of polycyclic aromatic hydrocarbons (PAHs). The dosages [300 (high dose), 30 (medium dose), or 3 (low dose) mg/kg body weight of NAFCs] were designed to reflect 50, 5, or 0.5 times, respectively, a worst-case, single-day exposure for wild mammals. Rats exposed to high doses of NAFCs exhibited significant metabolic changes, including a notable increase in hepatic glycogen storage and a small but significant reduction in body weight in males. Interestingly, the livers of NAFC-exposed rats were 36% heavier than those of controls, and this was accompanied by the presence of pericholangitis, an inflammation of the bile ducts observed in both male and female rats. These findings indicate that the liver is a primary target of NAFC toxicity, with structural and functional changes manifesting as increased organ weight and hepatic glycogen storage. Although glycogen storage is a typical metabolic function, excessive intracellular accumulation may indicate hepatotoxic effects or impaired carbohydrate metabolism [56]. Similarly, our study found changes in carbohydrate handling with a reduction in glucose uptake at both 24 and 48 h following 73.4 mg/L NAFC exposure. The observed decrease in glucose uptake suggests an early-stage metabolic adaptation, where the cells switch to alternative energy sources, such as fatty acids, to maintain energy homeostasis.

Altered utilization of glucose and triglycerides could have significant implications for organismal health and fitness. In mammals, efficient metabolic flexibility—the ability of the liver to switch between glucose and fatty acid utilization—is essential for maintaining energy homeostasis during periods of varying nutrient availability, such as times of abundance versus scarcity [57,58]. In our study, we observed decreased glucose uptake in association with evidence of increased mRNA expression of fatty acid beta-oxidation genes (i.e., *Cpt1α* and *Cd36)* in cells treated with 73.4 mg/L NAFCs. *Cpt1α* and *Cd36* are critical for the transport and oxidation of fatty acids. This suggests that, in response to reduced intracellular glucose uptake, hepatocytes may upregulate fatty acid oxidation pathways as an adaptive mechanism to sustain ATP production and overall energy homeostasis [59]. Indeed, during periods of nutrient scarcity, organisms typically break down fat stores (triglycerides reserves) into free fatty acids, which enter the circulation and are oxidized by metabolically active tissues [58,60]. Interestingly, fatty acid uptake decreased at 14.7 mg/L but increased at 73.4 mg/L, suggesting a dose-related response. The initial decrease at 14.7 mg/L could signal early mitochondrial dysfunction, while the increase at 73.4 mg/L may represent a compensatory mechanism for maintaining energy homeostasis in response to impaired glucose metabolism.

### 4.2. Mitochondrial Dysfunction as a ‘Switch’ for NAFC-Induced Metabolic Reprogramming

This shift towards fatty acid utilization, coupled with the increase in mitochondrial membrane potential (Δψm) at the 73.4 mg/L concentration, supports the hypothesis of mitochondrial hyperpolarization and stress. The continuous influx of fuel and incomplete utilization can lead to the accumulation of reducing equivalents within the mitochondria, increasing the mitochondrial membrane potential [57,61]. Elevated Δψm can enhance the production of reactive oxygen species (ROS), leading to oxidative stress, cellular damage, deleterious protein modifications, and ultimately metabolic reprogramming and disease [58,60].

In our study, we saw an increase in both ATP production and mitochondrial membrane potential at the same concentrations where we observed an increase in markers of fatty acid oxidation (i.e., Cpt1α and Cd36). While increased mitochondrial membrane potential allows for enhanced ATP synthesis, as we observed with NAFCs, at high ΔΨm the electron transport chain produces more reactive oxygen species that are known to cause oxidative damage to mitochondrial components, leading to apoptosis [61]. Indeed, we saw a concomitant increase in apoptosis at both 14.7 mg/L and 73.4 mg/L NAFC concentrations. In this study, the increase in ΔΨm and ATP production are contrary to other studies where NAFCs have been shown to reduce mitochondrial electron transport chain activity and mitochondrial membrane potential [62,63,64]. This discrepancy might be related to the differences in experimental models (rainbow trout mitochondria vs. rat hepatocytes) or the specific conditions of exposure. Additionally, the observed increase in mitochondrial membrane potential could indicate an initial compensatory response to NAFC-induced stress, where the mitochondria attempt to maintain ATP production despite underlying dysfunction. Moreover, both increased and decreased ATP and ΔΨm are indicative of mitochondrial dysfunction, which can have profound effects on cell function and survival [61]. Collectively, these studies suggest that the mitochondria are an important target of NAFC toxicity in multiple vertebrate species, including mammals.

Together, the switch from glucose to fatty acid oxidation observed in NAFC-treated hepatocytes likely reflects a form of metabolic compensation in response to impaired glucose uptake. This adaptation is mediated by mitochondrial flexibility; however, increased reliance on fatty acid oxidation requires increased mitochondrial function due to the higher number of reducing equivalents generated per molecule of substrate [60,65]. These excess reducing equivalents can lead to over-reduction in the electron transport chain, mitochondrial membrane hyperpolarization, and increased ROS production. Thus, while the elevated ATP production and ΔΨm may initially appear adaptive, they may in fact signal early mitochondrial stress. When sustained, this state can promote oxidative damage, trigger apoptosis, as observed in the TUNEL staining, and ultimately disrupt energy homeostasis. These findings support the view that NAFC-induced mitochondrial dysfunction may arise not only from direct toxicant interference but also from a maladaptive metabolic shift in substrate utilization. Indeed, oxidative stresses and subsequent cellular injuries can drive metabolic reprogramming, contributing to decreased growth rates and reduced overall fitness [58]. Importantly, this aligns with findings from Rogers et al. (2002), who reported decreased body weight and increased liver weights in rats exposed to high doses of NAs, indicative of hepatic stress and compromised growth [56].

### 4.3. Kynurenine–Tryptophan Ratio as a Marker of Metabolic Reprogramming

There is increasing recognition that environmental xenobiotics capable of altering glucose and lipid homeostasis may also influence tryptophan (TRP) metabolism [47], an essential amino acid involved in numerous physiological processes including growth and the synthesis of important biomolecules such as serotonin, melatonin, and niacin. For example, the prototypical AhR ligand, benzo[a]pyrene (BaP), has been shown to induce metabolic reprogramming alongside disruptions in amino acid metabolism in various mammalian cancer models [66,67,68]. A recent study investigating early life exposure to PCB126 in mice demonstrated acute alterations in hepatic metabolism, with significant reductions in TRP levels observed by the sixth day, although downstream KYN metabolites or KTR were not assessed [69]. In aquatic species, similar disruptions in tryptophan metabolism have been reported following exposure to naphthenic acids. For example, the common reed (*Phragmites australis*), when exposed to NAs, showed widespread metabolic alterations, including changes in amino acid metabolism, with tryptophan being identified as a significant marker of NA exposure (*p* = 0.022) [70]. Similarly, in a human leukemia monocytic cell line (THP1) exposed to OSPW [25% *v*/*v* OSPW in cell culture medium], the upregulation of kynureninase (KYNU)—an enzyme in the kynurenine pathway—was observed, further linking NA exposure to altered tryptophan metabolism [71]. While compounds that act as AhR ligands have been shown to affect tryptophan metabolism, other pathways have also been implicated including GR and peroxisome proliferator-activated receptor (PPAR) signaling, oxidative stress, and inflammatory pathways. Given that NAFCs are a complex chemical mixture, they likely interact with multiple signaling pathways to alter tryptophan metabolism, with some evidence suggesting limited AhR-mediated activity [72], GR signaling [43], and PPAR-related pathways [73]. Regardless, the changes in KTR, altered lipid and glucose metabolism, and mitochondrial function all occur in concert, suggesting that KTR may have utility as a hepatic marker of metabolic responses to NAFC exposure.

## 5. Conclusions

These findings provide valuable insights into how NAFCs may disrupt metabolic homeostasis in mammals. The observed metabolic reprogramming, including shifts in substrate utilization, mitochondrial dysfunction, and alterations in tryptophan metabolism, highlights a possible mechanism by which NAFCs impact the health of wildlife in the AOSR. The link between these cellular disruptions and reported cases of diminished growth and altered body condition in wildlife exposed to NAFCs suggests that these metabolic changes could be contributing to broader ecological consequences. By identifying the KTR as a potential biomarker of NAFC-induced metabolic alterations, this research offers a valuable tool for assessing the impact of bitumen-derived organic compounds on wildlife. Furthermore, these findings can be integrated into adverse outcome pathways (AOPs), which serve as structured frameworks that link molecular-level events to adverse biological outcomes. By mapping the metabolic and cellular disruptions caused by NAFCs onto established AOPs, this research contributes to the development of more robust environmental biomonitoring strategies. The ability to detect early molecular changes, such as metabolic reprogramming and KTR, allows for more proactive assessment of dynamic environmental conditions, including climate change. Therefore, this work not only advances our understanding of how NAFCs affect wildlife but also provides practical tools for environmental evaluation and protection.

## Figures and Tables

**Table 1:** Table of primer sequences.

Accession Number	Gene Name	Symbol	Forward Sequence (5′-3′)	Reverse Sequence (5′-3′)
NM_017101.1	Peptidylprolyl isomerase A	Ppia	CCGCTGTCTCTTTTCGCC	GCTGTCTTTGGAACTTTGTCTGC
NM_012512.2	Beta-2-microglobulin	B2m	AATTCACACCCACCGAGACC	GCTCCTTCAGAGTGACGTGT
NM_017332.1	Fatty acid synthase	Fasn	GGACATGGTCACAGACGATGAC	CGTCGAACTTGGACAGATCCTT
NM_022193.1	Acetyl-CoA carboxylase alpha	Acca	ATTGGGCACCCCAGAGCTA	CCCGCTCCTTCAACTTGCT
NM_031559.2	Carnitine palmitoyl transferase 1A	Cpt1a	CACCCCAACCCATATCCAGG	TCCTCACGGTCTAATGTGCG
NM_031561.2	Fatty acid translocase	Cd36	TCTCACACAACTCAGATACTGCT	GCACTTGCTTCTTGCCAACT
NM_013098.2	Glucose-6-phosphatase	G6pase	AACTCCAGCATGTACCGCAA	AAACGGAATGGGAGC GAC TT
NM_031347.1	Peroxisome proliferator-activated receptor-γ coactivator	Pgc1a	ATGGAGTGACATAGAGTGTGCT	CACCACTTCAATCCACCCAGA

## Data Availability

The original contributions presented in this study are included in the article. Further inquiries can be directed to the corresponding author.

## Abbreviations

The following abbreviations are used in this manuscript:

AOSR	Alberta Oil Sands Region
OSPW	Oil sands process-affected water
NAFC	Naphthenic acid fraction component
NA	Naphthenic acid
ATP	Adenosine triphosphate
TUNEL	Terminal deoxynucleotidyl transferase dUTP nick-end labeling
KTR	Kynurenine–tryptophan ratio
TRP-KYN	Tryptophan–kynurenine
PAC	Polycyclic aromatic compound
PAH	Polycyclic aromatic hydrocarbon
AhR	Aryl hydrocarbon receptor
GR	Glucocorticoid receptor
TRP	L-tryptophan
KYN	L-kynurenine
ΔΨm	Mitochondrial membrane potential
KYNU	Kynureninase
PPAR	Peroxisome proliferator-activated receptor
AOP	Adverse outcome pathway
